# Prevalence and risk factors associated with *Cryptosporidium* spp. infection in local breed of dogs in Enugu State, Nigeria

**DOI:** 10.14202/vetworld.2019.729-734

**Published:** 2019-05-31

**Authors:** Ukamaka U. Eze, Ikenna O. Ezeh, Terry A. Nzeakor, Samuel C. Attama, Ekene V. Ezenduka, Denchris N. Onah

**Affiliations:** 1 Department of Veterinary Medicine, Faculty of Veterinary Medicine, University of Nigeria, Nsukka, Nigeria; 2 Department of Veterinary Parasitology and Entomology, Faculty of Veterinary Medicine, University of Nigeria, Nsukka, Nigeria; 3 Department of Veterinary Public Health and Preventive Medicine, Faculty of Veterinary Medicine, University of Nigeria, Nsukka, Nigeria

**Keywords:** *Cryptosporidium*, dogs, modified Ziehl–Neelsen techniques, Nigeria, prevalence, risk factor

## Abstract

**Aims:**

Cryptosporidiosis is an important zoonotic disease of major public and veterinary concern. The disease affects humans and a variety of animal species including the domestic dog. This study aimed to determine the prevalence and risk factors associated with *Cryptosporidium* spp. infection in local breed of dogs from different homes and those presented at veterinary hospitals and clinics in Enugu State, Nigeria.

**Materials and Methods:**

A total of 203 fresh fecal samples were collected from domestic dogs in six local government areas in Enugu State from February 2015 to August 2015. All the samples were examined using the formol-ether sedimentation method. Fecal smears were then stained by the modified Ziehl–Neelsen technique and examined under direct light microscopy.

**Results:**

A total of 74 (36.5%) dogs were infected with *Cryptosporidium* spp. oocysts. There was a strong association (p<0.05) between the presence of *Cryptosporidium* spp. oocysts and management practices. However, there was no statistically significant association (p>0.05) between the presence of *Cryptosporidium* spp. oocysts and age, sex, and fecal consistency.

**Conclusion:**

The findings of this work suggest that domestic dogs in Enugu State harbor and shed *Cryptosporidium* spp. oocysts in the environment, especially those managed semi-intensively. Such fecal shedding is particularly so and of greater zoonotic and epidemiological importance in animals that do not show clinical signs and therefore not treated. They, therefore, pose a greater public health risk, especially to immune-compromised humans and animals. Public education on the zoonotic implication of this protozoan infection is of paramount importance in Enugu State, in particular, and Nigeria, in general, considering the closeness of dogs and man.

## Introduction

*Cryptosporidium* spp. is an apicomplexan protozoon of the order Eucocidiorida, subclass Coccidiansina, and class Sporozoasida [1]. This intracellular parasite affects the epithelial cell linings of the digestive system of a wide variety of mammalian hosts, including domestic dogs where they cause mucoid to bloody diarrhea and even death [2,3]. It is one of the most common intestinal protozoan parasites of animals and man, particularly in children and immunocompromised patients [4]. The parasite is the cause of large water-borne and food-borne outbreaks of gastroenteritis and is strongly associated with diarrhea, which may be severe in AIDS patients [4]. The close relationship between man and his domestic pets, particularly dogs, makes transmission of zoonotic diseases very easy, especially those that could be acquired through environmental contamination such as cryptosporidiosis. *Cryptosporidium canis* is the most frequently identified species of *Cryptosporidium* spp. in dogs and its isolation in human patients from both developed and under-developed countries suggests that it is zoonotic [3,5-7]. For instance, studies have shown that both dogs and children from the same household were infected with *C. canis*, further highlighting the zoonotic importance of the disease [8]. *Cryptosporidium* spp. have been shown, in a recent data by the global enteric multicenter study on the burden and etiology of childhood diarrhea in developing countries, to be the leading causes of moderate to severe diarrhea in children <2 years of age [9]. Furthermore, the resistant stage (oocyst) produced by *Cryptosporidium* spp. is stable and can survive for weeks to months in the environment. Experimental studies suggest that even a single oocyst carries some probability of causing infection [10]. Canine intestinal cryptosporidiosis can result in serious or even fatal enteritis with diarrhea which may be hemorrhagic and accompanied by tenesmus, vomiting, abdominal pain, and inappetence, resulting in poor health, impaired development, and colitis, especially in puppies and immunocompromised adult dogs, and death could result from excessive loss of electrolyte and dehydration [11-14].

In Nigeria, especially in rural areas, most dog owners have little or no chance of providing orthodox veterinary health care for their pets as a result of a dearth or complete lack of veterinarians. This situation results in domestic dogs harboring parasitic and other infections including zoonoses; thus, posing public health risks to humans, especially children who play around in the environments that are usually contaminated with stray dog feces [8,15].

The emergence of *Cryptosporidium* spp. as one of the leading causes of death in HIV/AIDS patients [16,17] has resulted in increased research on the prevalence of and infection caused by the parasite in domestic animals [18,19]. Despite the reported high prevalence of the infection in domestic animals [18-22], cryptosporidiosis is usually not included as a possible cause of gastroenteritis and therefore omitted in the routine differential diagnosis of gastroenteritis in domestic dogs. The importance of enteric protozoan infections in dogs and their public health implications cannot be overemphasized. However, there is a dearth of information and research on cryptosporidiosis in domestic dogs in Enugu State, Nigeria, irrespective of the fact that dog keeping is common and that Enugu State is one of the states with moderately high prevalence (approximately 4%) of HIV/AIDS in Nigeria [23].

This study was, therefore, designed to determine the prevalence and risk factors associated with the shedding of *Cryptosporidium* spp. oocysts in feces of dogs in Enugu State, Nigeria.

## Materials and Methods

### Ethical approval and informed consent

Consent was sought and was willingly granted by pet owners in the study area before their pet animals were included in the study. Free treatment was administered to clinically infected animals as an incentive to the owners for willingly allowing their animals to be used for the study. The experimental protocol was approved by the Experimental Animal Ethics Committee of the University of Nigeria, Nsukka.

### Study area

**Figure-1:**
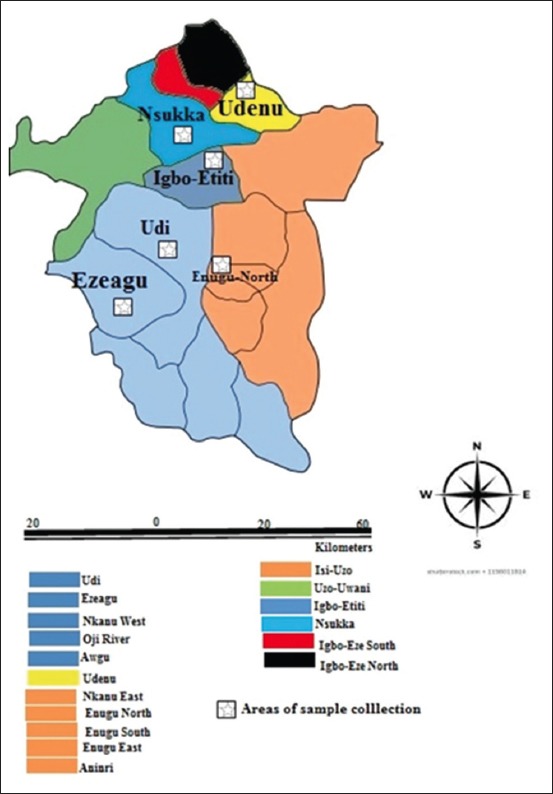
Map of Enugu showing areas of sample dogs collection.

The study was undertaken in Enugu State (Figure-1) which covers approximately 7617.82 km^2^ and is located between latitude 6° 45^’^ and 7°N and longitude 7°12.5^’^ and 7°36^’^ W in the Southeast geopolitical zone of Nigeria. It has three senatorial zones and 17 local government areas (LGAs). Simple random sampling technique was used to select two senatorial zones – Enugu West and Enugu North of the three senatorial zones in Enugu State. Samples were then collected from dogs from similarly randomly selected homes, veterinary hospitals, and clinics in three randomly selected LGAs in the two senatorial zones. Geographic positioning system was used to record the coordinates of the study area; thereafter, Geographic Information System – ArcView 3.3 [24] was used to construct the map showing areas of sample collection.

### Sample collection

Fresh fecal samples were collected from January to August 2015 from the randomly selected homes of dog owners, veterinary hospitals, and clinics. A total of 203 dogs were sampled in this study. Fecal samples were collected per rectum from each dog using a well-lubricated gloved finger into pre-labeled plastic containers. Animal identity including sex, age, and source was recorded for each dog. The management practices were graded as intensive for those dogs that stay in kennels and reside in a fenced compound and can only move around in the compound when released, while those that stay in kennels or chains but reside in compounds that are not fenced and can move around in the neighborhood when released were regarded as semi-intensive.

### Parasitological analysis

All stool samples were examined macroscopically, and their characteristics and consistency were recorded. The fecal consistencies were then categorized as formed, pasty or watery (diarrheic). The stool samples were then preserved at room temperature (25-28°C) in 5% formol saline until analyzed. The samples were concentrated by the formalin-ethyl acetate sedimentation technique [25], and then, thin smears of the sediments were made on glass slides, air-dried, and fixed with methanol. For the detection of *Cryptosporidium* spp. oocysts, modified Ziehl–Neelsen staining was performed, and the slides were examined using the oil immersion objective at 1000×. For quality control, all examinations were repeated twice by two experienced microscopists. The *Cryptosporidium* spp. was identified based on the oocysts staining characteristics.

### Statistical analysis

Results were analyzed by descriptive statistics and presented as percentages using tables and pie charts. GraphPad Prism statistical package version 5.2 for Windows (GraphPad Software, La Jolla, California, USA, www.graphpad.com) was used to analyze generated data, where Chi-square test was used to determine the association between the presence of *Cryptosporidium* spp. oocysts and management practice; presence of *Cryptosporidium* spp. oocysts and sex; and presence of *Cryptosporidium* spp. oocysts and fecal consistency. Odds ratio was used to determine the strength of association between the variables. Statistical significance was accepted at p<0.05.

## Results

**Figure-2:**
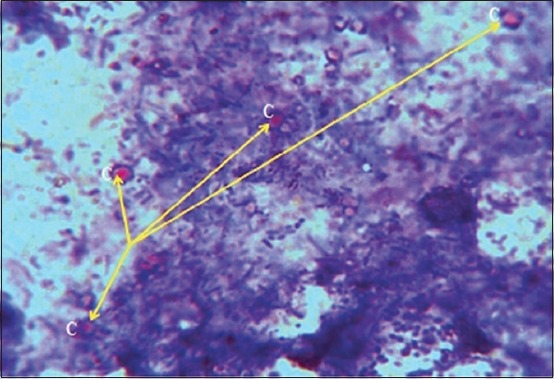
*Cryptosporidium* spp. oocysts (C=arrows) stained by modified Ziehl–Neelsen technique (1000×).

In positive samples, *Cryptosporidium* spp. oocysts were seen as red staining round bodies (Figure-2) about 5-8 µm against a blue background.

### Distribution of the parasite oocysts according to localities

Of the 203 fecal samples, 74 (36.5%) were positive for intestinal protozoan parasites. The distribution of the positive samples according to the LGAs was 22 (29.7%), 15 (20.3%), 11 (14.9%), 10 (13.5%), 9 (12.2%), and 7 (9.5%) for Udenu, Igbo-Etiti, Ezeagu, Udi, Nsukka, and Enugu North LGAs, respectively (Table-1).

**Table-1:** Distribution of *Cryptosporidium* spp. oocysts in six local government areas of Enugu State.

Local government area	Fecal samples (n)	Positive (n)	Percentage
Udenu	61	22	36.1
Igbo-Etiti	34	15	44.1
Ezeagu	24	11	45.8
Udi	20	10	50.0
Nsukka	44	9	20.5
Enugu North	20	7	35.0
Total	203	74	36.5

### Prevalence of *Cryptosporidium* spp. in fecal samples of dogs and their associated risk factors

**Figure-3:**
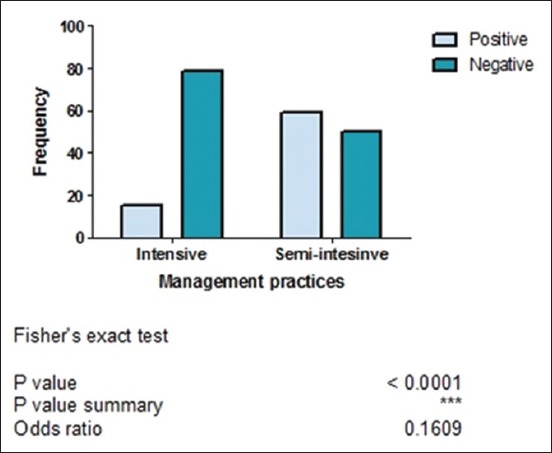
Association between the presence of *Cryptosporidium* spp. and management practices of sampled dogs in Enugu State, Nigeria.

**Figure-4:**
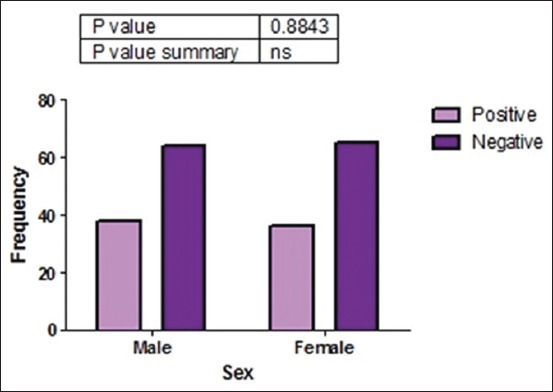
Association between the presence of *Cryptosporidium* spp. and sex of sampled dogs in Enugu State, Nigeria.

**Figure-5:**
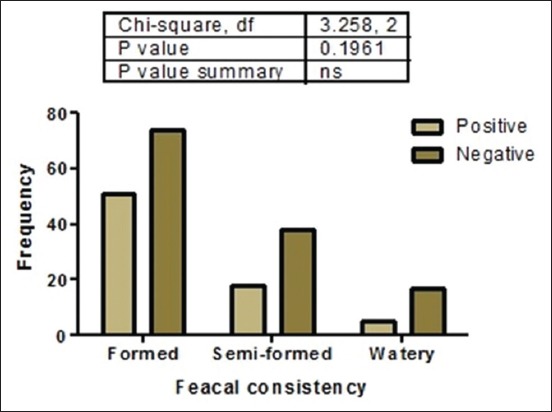
Association between the presence of *Cryptosporidium* spp. and fecal consistency of the feces of dogs sampled in Enugu State, Nigeria.

Of 203 fecal samples examined, 74 (36.5%) contained oocysts of *Cryptosporidium* spp. There was a strong association (p<0.05) between the presence of *Cryptosporidium* spp. oocysts and management practice (Figure-3). This study was done in rural areas where most people do not have access to good toilet facilities and they defecate in the bushes around residential buildings. Dogs managed semi-intensively roam the neighborhood, defecate indiscriminately, and equally have access to human feces in the environment. This behavior encourages contamination of the environment by both man and dogs and thus increases the chances of naïve dogs getting infected with *Cryptosporidium* spp. and probably other gastrointestinal parasites. Statistical analysis showed that there was no significant association (p>0.05) between the presence of *Cryptosporidium* spp. oocysts and sex (Figure-4). Furthermore, there was no significant association between the presence of *Cryptosporidium* spp. oocysts and fecal consistency (Figure-5). Therefore, the greatest risk factor for the maintenance and transmission of cryptosporidiosis in the study area was free roaming of dogs and open-air defecation by humans and dogs.

## Discussion

The prevalence of *Cryptosporidium* spp. oocysts in feces of local dogs in Enugu State, Nigeria, was determined in this study. An overall high prevalence of 36.5% was recorded. High prevalence of cryptosporidiosis has also been recorded in other developing nations such as Costa Rica (75%) [26] and South Africa (44%) [22]. However, the high prevalence recorded in this study differed from the prevalence of 5.8% obtained in dogs in Abuja, the capital of Nigeria [18]. This low prevalence in Abuja is similar to what was obtained in the developed countries such as the United States (3.8%), Australia (9.4%), and Japan (3.9%) [27-29]. Furthermore, the prevalence of *Cryptosporidium* spp. obtained in China (3.8%), Iran (8%), and Zambia (5.9%) is as low as those in developed countries [7,19,30]. The difference in the prevalence may be attributed to geographic location and standard of living, which determines the health care given to the pets. The level of education of pet owners, the reduced environmental contamination by dog’s feces, as well as proper sewage disposal of human waste and efficient public refuse disposal systems help reduce the level of environmental contamination by the parasite oocysts and, hence, the low prevalence. In this study, we sampled dogs in rural areas where dogs are allowed to roam with little provision of veterinary health care and welfare, hence, the high prevalence.

**Figure-6:**
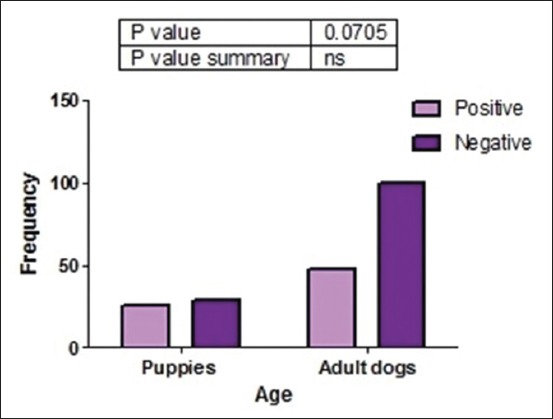
Association between the presence of *Cryptosporidium* spp. and age of dogs sampled in Enugu State, Nigeria.

In this study, we observed a strong association between the presence of *Cryptosporidium* spp. oocysts and management practice. It was observed that dogs managed semi-intensively had more *Cryptosporidium* spp. oocysts than those managed intensively. This is expected since those dogs’ roam and thus are more predisposed to acquiring infections from the environment contaminated with human and dog feces. There was no statistically significant association between the presence of *Cryptosporidium* spp. oocysts and the age of dogs, although a higher prevalence of the parasite oocysts was seen in puppies than in adult dogs (Figure-6). This finding is in contrast with those of Olabanji *et al*. [18] and Mugala *et al*. [19] who reported that older dogs were more parasitized than puppies. Adults are said to have an increased tendency to roam and acquire the infection than puppies that have more restricted movement. However, our findings agree with those of Ramirez *et al*. [31], Hamnes *et al*. [32], and Jian *et al*. [7] who reported that puppies were more infected with *Cryptosporidium* spp. than adults. This may be due to immune incompetence in puppies, unlike older dogs.

Similarly, sex did not influence *Cryptosporidium* spp. infection as there were no significant associations (p>0.05) between them, although there was a higher prevalence of *Cryptosporidium* spp. oocysts in male than female dogs which could be attributed to the fact that males move around more widely than females in search of mating partners and establishment of territory. This activity predisposes them to various kinds of infections, including intestinal protozoan infection. However, other workers reported higher infection in females than in males and attributed it to reduced immunity at certain periods in the female’s physiologic cycle [18,33,34].

Oocysts were seen more in apparently healthy dogs (40%) than those having signs of enteritis (diarrhea) – 22% (watery feces) and 32% (semi-solid feces). It appeared that the more the feces was formed, the higher the chances of presence of oocysts. Diarrhea is indicative of acute clinical cryptosporidiosis occasioned by the activities of the sporozoites and merozoites in the intestinal epithelia. Therefore, sufficient oocysts may not have been produced at the diarrheic phase to be shed in feces, especially as it has been observed that oocyst shedding in dogs infected with *Cryptosporidium* spp. occurs when the mature oocysts are formed following a successful asexual cycle [35]. This may have explained the reason for finding rather higher positive cases in formed feces. Our findings, in this work, are also in agreement with previous reports indicating that most infections in dogs are asymptomatic [7,19,31]. However, it contrasts with findings from other workers who reported seeing more oocysts in diarrheic feces [18,20,21], particularly in young animals [36].

The locality had no significant association (p>0.05) with *Cryptosporidium* spp. infection. Nonetheless, Udenu had the highest (25%) prevalence, followed by Igbo-Etiti (20%). This may be because these areas are rural communities and have a higher density of dogs which are allowed to roam, the access to veterinary health care is low, and therefore these dogs are likely to have easy access to these oocysts from the environment. Therefore, dogs in such areas are predisposed to a high risk of contracting infections.

## Conclusion

This study has established a high prevalence of *Cryptosporidium* spp. oocysts in domestic dogs in Enugu State, Nigeria. The rate of infection was higher in non-diarrheic and semi-intensively managed dogs. The presence of oocysts in feces by apparently healthy and free-roaming dogs poses public health risk to humans, immune-compromised adult animals, and puppies. Therefore, public health education and awareness of the zoonotic importance of these enteric protozoan parasites are imperative among dog owners and the general public. The infection should also be included in the list of routinely diagnosed diseases in veterinary hospitals and clinics as a possible cause of enteritis and diarrhea, as seen in this study.

## Authors’ Contributions

UUE and IOE conceived the work; TAN, SCA, and EVE collected and analyzed the samples; EV statistically analyzed the generated data; IOE, UUE, and DNO wrote, reviewed, and edited the manuscript. All authors read and approved the final manuscript.
